# The Impact of Maternal Risk Factors on Neonatal Morbidity and Mortality in a Tertiary Care Neonatal Intensive Care Unit (NICU): An Observational Study

**DOI:** 10.7759/cureus.65714

**Published:** 2024-07-29

**Authors:** Shailesh Wandile, Manoj Waghmode, Punam Uke, Jayant D Vagha, Chaitanya Kumar Javvaji, Ajinkya Wazurkar

**Affiliations:** 1 Pediatrics, Jawaharlal Nehru Medical College, Datta Meghe Institute of Higher Education and Research, Wardha, IND; 2 Pediatrics and Neonatology, Government Medical College, Aurangabad, IND

**Keywords:** high-risk mothers, maternal risk factors, neonatal intensive care unit (nicu), morbidity, mortality

## Abstract

Background: Neonatal morbidity and mortality continue to be major public health issues globally, especially for infants admitted to neonatal intensive care units (NICUs). This study aims to investigate the incidence of morbidities among neonates born to high-risk mothers and to evaluate the impact of various maternal risk factors on neonatal morbidity and mortality in the NICU setting.

Methods: This prospective observational study was conducted on 1,000 newborns up to 28 days of life, all with maternal risk factors, born in our tertiary care center, and admitted to the NICU.

Results: Most NICU admissions occurred during the 34-36 weeks of gestation, comprising 412 (41.20%) of the total admissions. Additionally, there was a female predominance, with 552 cases, representing 55.20% of the admissions. Most of the NICU patients came from rural background 594 (59.40%) and belonged to socioeconomic status (SES) IV 764 (76.40%). Higher percentages of neonatal morbidities were observed among children of illiterate and primarily educated mothers, amounting to 913 cases (91.30%). After evaluating patients in the NICU, we found that mortality was 172 (17.20%). Mothers with previous bad obstetric histories were at greater risk of poor neonatal outcomes. Cesarean sections were more commonly associated with NICU admissions, accounting for 555 cases (55.50%). The primary risk factors included pregnancy-induced hypertension, previous lower segment cesarean section, fetal distress, and premature rupture of membranes. Significant neonatal morbidities included respiratory distress syndrome (RDS) due to prematurity 79 (45.9%), intrauterine growth retardation 19 (11.0%), meconium aspiration syndrome 16 (9.3%), birth asphyxia, sepsis 29 (16.8%), and congenital anomalies 12 (6.9%). RDS was identified as the leading cause of morbidity.

Conclusion: The present study highlights several critical factors associated with NICU admissions and neonatal morbidities, underscoring the need for targeted interventions to improve neonatal health outcomes.

## Introduction

The neonatal period encompasses the first 28 days of life and is the most vulnerable time for mortality and morbidity. The health status of a country is often assessed through its infant mortality rate, with neonatal mortality contributing to two-thirds of this figure. In India, the current neonatal mortality rate (NMR) is 34 per 1,000 live births. Notably, 75% of neonatal deaths occur within the first week of life. The primary causes of neonatal mortality in developing countries, accounting for 78% of cases, include prematurity and low birth weight, neonatal infections, and birth asphyxia. Following the reduction in NMR, the focus shifts to decreasing neonatal morbidity through targeted disease-specific interventions. Therefore, understanding the pattern of medical illnesses in a specific region is crucial for healthcare providers to plan and prioritize services effectively [1].

Pregnancy at high risk refers to pregnancy followed by variables that raise the risk of morbidity and neonatal mortality. Based on statistical data, 10%-20% of pregnancies are recorded as high-risk pregnancies. Neonatal well-being has a significant influence on future health and life. Because the immune system of neonates and other organs in preterm neonates are not fully established, there are more chances of neonatal admission to the neonatal intensive care unit (NICU) for a short or long period in the first month of life [2].

Previous studies have indicated that preterm infants face a higher risk of both acute and long-term health issues, which, in turn, affects their NICU stay duration compared to full-term infants [3,4]. Another significant clinical complication, premature rupture of membranes (PROM), is linked with elevated neonatal morbidity and mortality rates. Increased rates of morbidity and mortality have also been reported in late preterm infants born to women with gestational hypertension or preeclampsia, with these infants experiencing more frequent NICU admissions, hypoglycemia, respiratory failure, and rehospitalization [5].

Maternal gestational diabetes mellitus (GDM) is another condition associated with higher prenatal morbidity. Infants born to mothers with GDM are more likely to experience neonatal hypoglycemia, respiratory distress syndrome (RDS), hyperbilirubinemia, being large for gestational age (LGA), congenital disorders, primary cesarean section, polyhydramnios, and preterm birth. NICU admission rates are 29% for pregnancies complicated by GDM and 40% for those complicated by type 2 diabetes mellitus. Additionally, a more extended NICU stay is observed in pregnancies complicated by both hypertension and diabetes [2].

This study examined the impact of pregnancy complications on the length of stay for neonates in the NICU. Providing NICU care for newborns with complications imposes a significant burden on the healthcare system. While numerous studies have explored the factors leading to neonatal admission in the NICU, there is limited information on how maternal complications affect the duration of neonatal hospitalization. Investigating this relationship can help health organizations develop effective strategies to reduce high-risk pregnancies and, consequently, the length of neonatal NICU stays. Implementing such strategies would improve antenatal outcomes and significantly reduce healthcare costs.

## Materials and methods

Study design, setting, study period, and study participants

This prospective, observational study was conducted in the Department of Pediatrics at a tertiary care center from July 2018 to August 2020, following the approval from the Institutional Ethical Committee and obtaining written informed consent from all parents or guardians. This study was conducted on 1,000 newborns up to 28 days of life, all with maternal risk factors, born in our tertiary care center, and admitted to the NICU. Neonates without maternal risk factors and born outside our tertiary care center were excluded from the study.

Study procedure

All data were collected from the medical records of neonates and their mothers. The target population included eight to ten newborns admitted to the NICU daily. A checklist was used to record neonatal gestational age, sex, newborns' health issues, and the duration of NICU stay (days). Simultaneously, maternal obstetric medical records were reviewed to gather information on maternal complications such as PROM, preeclampsia, urinary tract infection, GDM, vaginal bleeding, and addiction.

We then statistically evaluated the impact of these complications on the length of neonatal NICU admission. Additional social demographic parameters were recorded, including age, gender, place of residence, educational status of the head of the family, and socioeconomic status (SES). Data about mothers and infants were collected using a checklist. This included information about neonatal age, birth weight, gender, maternal diseases, mode of delivery, causes and duration of hospitalization, complications during the stay, surfactant administration, use of mechanical ventilation, type of treatment, and disease outcomes.

Statistical analysis

The data analysis was performed using SPSS IBM version 21.0 (IBM Corp., Armonk, NY). Both univariate and bivariate analyses were conducted. For qualitative variables, proportions were calculated, while quantitative variables were analyzed using mean ± SD. Relevant tests of significance, such as chi-square tests and independent t-tests, were applied. A p value of <0.05 was considered statistically significant.

## Results

The majority of the neonates were born between 34 and 36 weeks of gestational age, i.e., 412 (41.20%). The female predominance was seen in the study, accounting for 552 cases (55.20%). Most NICU patients came from rural backgrounds, i.e., 594 cases (59.40%), and belong to SES class IV, i.e., 764 cases (76.40%). In most cases, the head of the family's education level was up to primary school, accounting for 913 cases (91.30%). Mothers admitted to the NICU were mainly between the ages greater than 19 and less than 35, i.e., 889 cases (88.90%). Maximum patients, i.e., 752 cases (75.20%), belonged to appropriate for gestational age (AGA) birth weight (Table 1).

**Table 1:** Sociodemographic data of the subjects SGA: small for gestational age; AGA: appropriate for gestational age; LGA: large for gestational age

Sociodemographic data		Frequency	Percentage
Gestational age (weeks)	>37	168	16.80
	34-36	412	41.20
	31-33	308	30.80
	28-30	89	8.90
	25-27	19	1.90
	22-24	03	0.30
Mean ± SD	35.34 ± 3.64		
Gender	Male	448	44.80
	Female	552	55.20
Geographical area	Rural	594	59.40
	Urban	406	40.06
Socioeconomic status	I	00	0.00
	II	06	0.60
	III	198	19.80
	IV	764	76.40
	V	32	3.20
Educational status of the head of the family	Illiterate	165	16.50
	Primary education	748	74.80
	Secondary and higher secondary	41	4.10
	Graduate or higher	46	4.60
Maternal age (years)	≤19	84	8.40
	>19 to <35	889	88.90
	≥35	27	2.70
Birth weight	SGA	225	22.50
	AGA	752	75.20
	LGA	23	2.30

**Figure 1:**
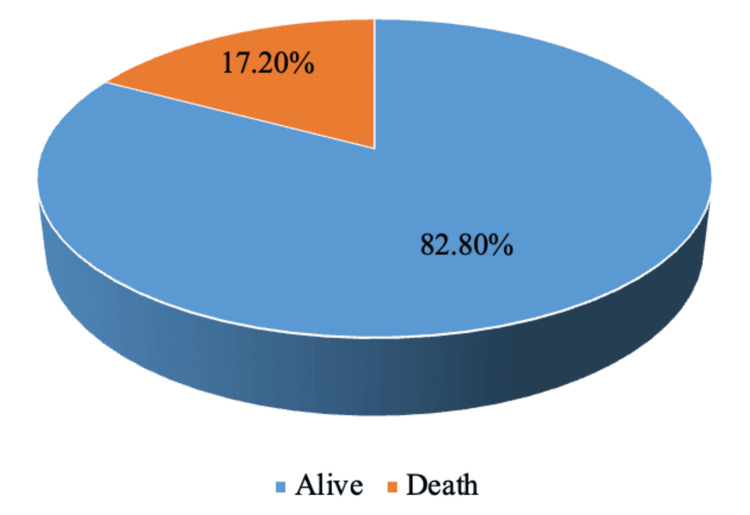
Outcome of NICU admissions in the study period NICU: neonatal intensive care unit

After evaluating patients in the NICU, we found a mortality rate of 17.20%, which amounts to 172 cases, as depicted in Figure 1.

The majority of deaths occurred in SES class V, i.e., 12 cases (37.50%), followed by class III, i.e., 42 cases (21.21%). Both extremes show a peak of mortality, i.e., ≥35 years, representing 55.55% of 15 cases, followed by 19 years with 54.76% of 46 cases. As expected, death was also found in mothers who lack antenatal care, i.e., 55 cases (44.69%). The mortality rate for individuals with a history of previous bad obstetrics was 36 cases (17.91%). Mothers with a bad obstetric history were found to be 201 (20.10%) (Table 2).

**Table 2:** Sociodemographic profile versus outcome

Parameters		Alive	Death	p value
Socioeconomic status	I	0 (0%)	0 (0%)	0.001877
	II	6 (100.0%)	0 (0%)	
	III	156 (78.89%)	42 (21.21%)	
	IV	646 (84.56%)	118 (15.44%)	
	V	20 (62.50%)	12 (37.50%)	
Maternal age (years)	≤19	38 (45.24%)	46 (54.76%)	<0.00001
	>19 to <35	778 (87.52%)	111 (12.48%)	
	≥35	12 (45.45%)	15 (55.55%)	
Antenatal care	Received	755 (86.99%)	113 (13.01%)	<0.00001
	Not received	73 (55.31%)	59 (44.69%)	
Previous bad obstetrics history	Yes	165 (82.09%)	36 (17.91%)	0.7652
	No	663 (82.98%)	136 (17.02%)	

**Figure 2:**
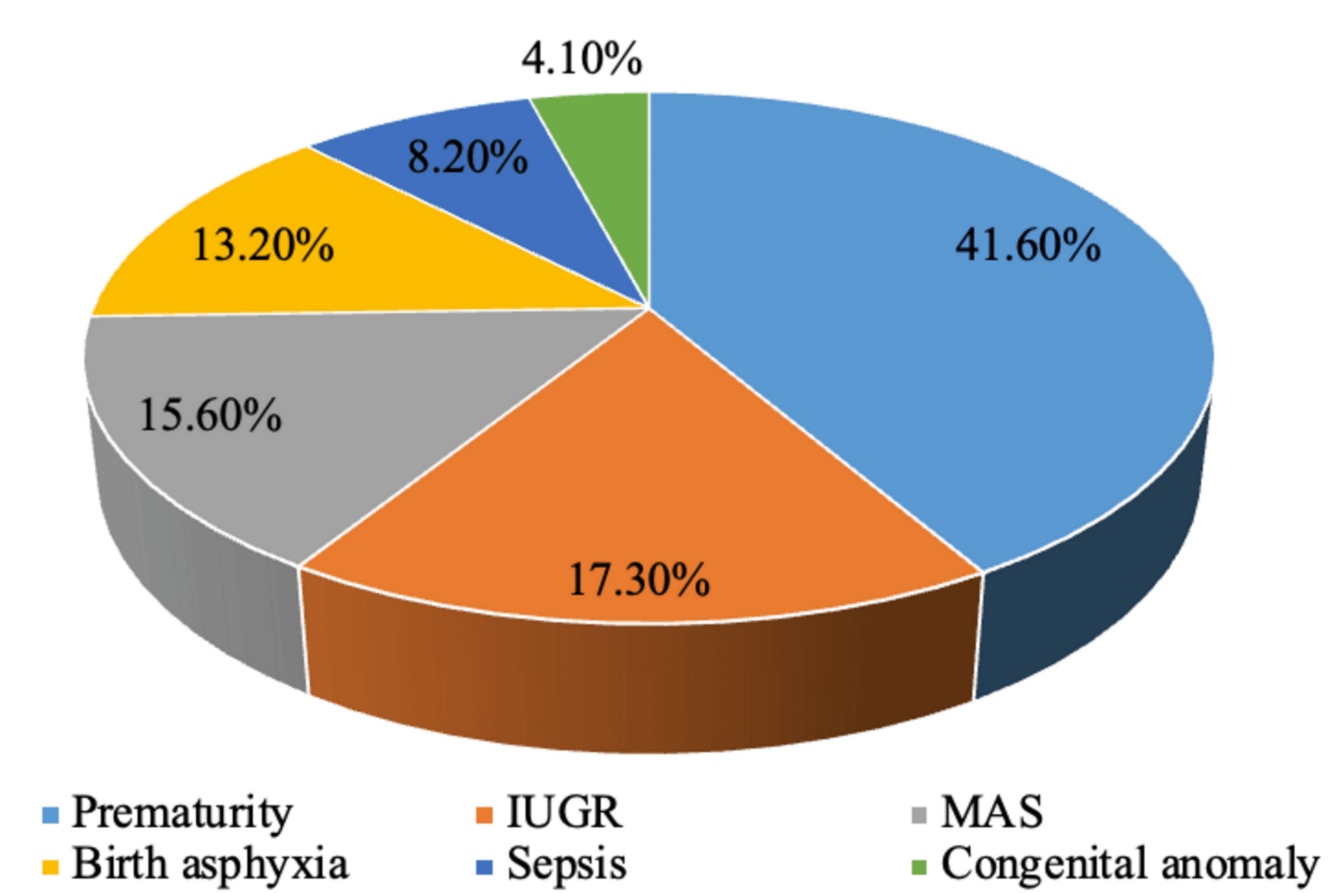
Distribution according to causes of NICU admission MAS: meconium aspiration syndrome; IUGR: intrauterine growth restriction; NICU: neonatal intensive care unit

Most NICU admissions were due to prematurity, i.e., 416 (41.60%), followed by intrauterine growth retardation (IUGR), 173 (17.30%), and meconium aspiration syndrome (MAS), 156 (15.60%), respectively, as depicted in Figure 2.

When comparing gestational age to the outcome, we found that 100% of deaths occurred between 22 and 24 weeks (three cases), 100% between 25 and 27 weeks (19 cases), and 65.16% between 28 and 30 weeks (58 cases). Most of the deaths were from the female gender, i.e., 93 (16.84%), as the family is most small for gestational age (SGA), i.e., 110 (48.88%). Most NICU admissions were after cesarean section, i.e., 550 (55.50%). Most percentages of death were from vaginal delivery, i.e., 81 (18.20%). The primary cause of a cesarean section was found to be pregnancy-induced hypertension (PIH), i.e., 219 (39.41%), followed by previous lower segment cesarean section (LSCS), i.e., 125 (22.52%). Most of the deaths were caused by sepsis, accounting for 29 (35.36%) of the cases, followed by congenital anomalies at 12 (29.26%) (Table 3).

**Table 3:** Neonatal parameters versus outcome SGA: small for gestational age; AGA: appropriate for gestational age; LGA: large for gestational age; NICU: neonatal intensive care unit; IUGR: intrauterine growth restriction; MAS: meconium aspiration syndrome

Parameters		Alive	Death	p value
Gestational age (weeks)	>37	148 (88.10%)	20 (11.90%)	<0.00001
	34-36	391 (94.91%)	21 (5.09%)	
	31-33	257 (83.45%)	51 (16.55%)	
	28-30	31 (34.84%)	58 (65.16%)	
	25-27	00 (0.0%)	19 (100.0%)	
	22-24	00 (0.0%)	3 (100.0%)	
Mode of delivery	Vaginal	364 (81.80%)	81 (18.20%)	0.4520
	Cesarean section	464 (83.61%)	91 (16.39%)	
Gender	Male	369 (82.37%)	79 (17.63%)	0.7432
	Female	459 (83.16%)	93 (16.84%)	
Birth weight	SGA	115 (51.12%)	110 (48.88%)	<0.00001
	AGA	690 (91.76%)	62 (8.24%)	
	LGA	23 (100.0%)	0 (0%)	
NICU admission	Prematurity	337 (81.01%)	79 (18.99%)	<0.00001
	IUGR	154 (89.02%)	19 (10.98%)	
	MAS	140 (89.75%)	16 (10.25%)	
	Birth asphyxia	115 (87.12%)	17 (12.87%)	
	Sepsis	53 (64.64%)	29 (35.36%)	
	Congenital anomaly	29 (70.74%)	12 (29.26%)	

Most deaths were due to heart disease, i.e., two cases (40%), followed by cephalopelvic disproportion (CPD) and prolonged labor, as shown in Table 4.

**Table 4:** Distribution of perinatal morbidity according to maternal risk factors vs. outcome PIH: pregnancy-induced hypertension; PROM: premature rupture of membranes; CPD: cephalopelvic disproportion; APH: antepartum hemorrhage; GDM: gestational diabetes mellitus

Maternal risk factors	Total	Alive	Death	p value
PIH	358	290 (81.01%)	68 (18.99%)	0.2938
Premature labor	327	281 (85.94%)	46 (14.06%)	
Severe anemia	74	62 (83.79%)	12 (16.21%)	
Multiple pregnancies	46	38 (82.61%)	8 (17.39%)	
PROM	40	31 (77.50%)	9 (22.50%)	
CPD	34	23 (97.65%)	11 (32.35%)	
APH	34	31 (91.18%)	3 (8.82%)	
Abnormal lie	27	21 (77.78%)	6 (22.22%)	
Prolonged labor	17	12 (70.59%)	5 (29.41%)	
Oligohydramnios	17	15 (88.24%)	2 (11.76%)	
Heart disease	5	3 (60.0%)	2 (40.0%)	
GDM	5	5 (100.0%)	0 (0%)	
Others, e.g., renal insufficiency	16	16 (100.0%)	0 (0%)	

## Discussion

In the present research, most neonates were born at a gestational age between 34 and 36 weeks, accounting for 412 cases (41.20%). The lowest number of neonates was born at a gestational age between 22 and 24 weeks, amounting to three cases (0.30%). This finding is similar to the prior studies [6-8]. During the study period, the NICU had a higher admission rate for female patients, with 552 admissions, accounting for 55.20% of the total sample. Other studies indicated a higher male-gender ratio in NICU admissions [7,8]. Most of the NICU patients came from rural backgrounds, 594 (59.40%), and belonged to socioeconomic status III 198 (19.80%) and IV 764 (76.40%). Most mothers had only received education up to the primary school level, 748 (74.80%), followed by 165 (16.50%) who were illiterate.

Similar findings are reported in the study done by Kotwal et al. [9]. Maximum NICU admission mothers belonged to the age group of greater than 19 to less than 35 years, 889 (88.90%), which is comparable with the study conducted by Shetty et al. [6]. Eight out of 117 neonates born to mothers with a height of less than 140 cm did not survive, i.e., 6.83%, and the number of neonatal deaths for babies born to mothers weighing less than 40 kg was 38 out of 205, i.e., 18.53%. The results are very similar to the study done by Agrawal and Bhatnagar [10]. In our study, the birth weights were predominantly AGA at 752 cases (75.20%), followed by SGA at 225 cases (22.50%). These findings are consistent with those reported by Jeganathan et al. [11] and Gogoi [12].

After evaluating patients in the NICU, we found a mortality of 172 (17.20%), which is correlated with the study done by Kotwal et al. [9] and Jeganathan et al. [11]. Comparing gestational age with outcomes, we found that the highest percentage of deaths occurred at 22-24 and 25-27 weeks, both at 100%, followed by 28-30 weeks, i.e., 58 (65.16%). The majority of deaths were among females, 93 (16.84%). Most deaths were in the SGA category, 110 (48.88%), followed by AGA 62 (8.24%), and no deaths in the LGA category.

There was a peak of mortality at both extremes, i.e., ≥35 years, 15 (55.55%), followed by ≤19 years, 46 (54.76%), and between 19 and 35 years, 111 (12.48%), which was comparable with the study done by Philip and Pramod [13]. Despite implementing numerous government schemes, some mothers did not receive antenatal care. In the current study, we found this to be the case for 132 mothers (13.20%), which is consistent with the findings of Agrawal and Bhatnagar [10]. Mothers having a bad obstetric history were found to be 201 (20.10%), while those without a bad obstetric history were 799 (79.90%).

Mortality on a previous bad obstetric history was 36 (17.91%). The results were very similar to the study conducted by Philip and Pramod [13] and Naskar et al. [14]. Most NICU admissions were after cesarean section, totaling 555 (55.50%) [13,14]. The leading cause of cesarean section was PIH, 358 (35.8%), followed by a history of previous LSCS, which is consistent with the findings of the study by Shetty et al. [6]. The majority of deaths occurred as a result of vaginal delivery, accounting for 81 cases (18.20%). Most NICU admissions were due to prematurity, specifically RDS, i.e., 416 (41.60%), followed by IUGR and MAS, i.e., 173 (17.30%) and 156 (15.60%), respectively. Pengoria et al. reported that 322 cases (65.8%) had RDS, 20 cases (1.4%) developed seizures, and five cases (1%) had sepsis [15].

The present study did not have a higher percentage of physiological jaundice because the study included patients with maternal risk factors, so other serious illnesses were more, and there need not be any specific risk factor for physiological jaundice to occur. Most percentage of the deaths were caused by sepsis, followed by congenital anomaly and RDS. These findings are in accordance with the study conducted by Anurekha et al. [8], Naskar et al. [14], and Pengoria et al. [15].

The neonatal morbidities were as follows: PIH: 358 (35.80%), premature labor: 327 (32.70%), severe anemia: 74 (7.40%), multiple pregnancies: 46 (4.60%), PROM: 40 (4.00%), CPD: 34 (3.40%), antepartum hemorrhage (APH): 34 (3.40%), abnormal lie: 27 (2.70%), prolonged labor: 17 (1.70%), oligohydramnios: 17 (1.70%), heart disease: 5 (0.50%), and GDM: 5 (00.50%). However, the details of neonatal deaths are as follows: PIH: 68 (18.99%), premature labor: 46 (14.06%), severe anemia: 12 (16.21%), multiple pregnancies: 8 (17.39%), PROM: 9 (22.50%), CPD: 11 (32.35%), APH: 3 (8.82%), abnormal lie: 6 (22.22%), prolonged labor: 5 (29.41%), oligohydramnios: 2 (11.76%), heart disease: 2 (11.76%). These findings are comparable with the previous studies done by Shetty et al. [6], Kambiakdik et al. [7], Philip and Pramod [13], and Naskar et al. [14].

## Conclusions

The study highlights several critical factors linked to NICU admissions and neonatal morbidities, emphasizing the need for targeted interventions to improve neonatal health outcomes. Key risk factors identified include PIH, previous caesarean sections, fetal distress, and PROM. Major neonatal morbidities such as RDS due to prematurity, IUGR, MAS, birth asphyxia, sepsis, and congenital anomalies were noted, with RDS being the leading cause. Low birth weight and prematurity significantly contributed to neonatal mortality, underscoring the importance of strengthening antenatal programs. Additionally, the high incidence and mortality associated with birth asphyxia call for effective prevention and management strategies. Addressing these multifaceted issues through comprehensive healthcare and educational interventions is crucial for reducing NICU admissions and enhancing overall neonatal health outcomes.

## References

[1] Kanimozhi P, Kumaravel KS, Velmurugan K (2019). A study on the length of stay of neonates in neonatal intensive care unit in a referral hospital in India. Int J Contemp Pediatrics.

[2] Afrasiabi N, Mohagheghi P, Kalani M, Mohades G, Farahani Z (2014). The effect of high risk pregnancy on duration of neonatal stay in neonatal intensive care unit. Iran J Pediatr.

[3] Bastek JA, Sammel MD, Paré E, Srinivas SK, Posencheg MA, Elovitz MA (2008). Adverse neonatal outcomes: examining the risks between preterm, late preterm, and term infants. Am J Obstet Gynecol.

[4] Sengupta S, Carrion V, Shelton J, Wynn RJ, Ryan RM, Singhal K, Lakshminrusimha S (2013). Adverse neonatal outcomes associated with early-term birth. JAMA Pediatr.

[5] Habli M, Levine RJ, Qian C, Sibai B (2007). Neonatal outcomes in pregnancies with preeclampsia or gestational hypertension and in normotensive pregnancies that delivered at 35, 36, or 37 weeks of gestation. Am J Obstet Gynecol.

[6] Shetty MB, Krupa B, Malyala M, Swarup A, Pathadan DS, Pocha S (2017). Preterm birth: associated risk factors and outcome in tertiary care center. Int J Reprod Contracept Obstetr Gynecol.

[7] Kambiakdik T, Leelalanslat AD, Sohi I, Varkey VP (2018). Maternal risk factors and early neonatal outcome among late preterm and term neonates in a neonatal intensive care unit in Punjab, India. Int J Contemp Pediatrics.

[8] Anurekha V, Kumaravel KS, Kumar P, Satheesh Kumar D (2018). Clinical profile of neonates admitted to a neonatal intensive care unit at a referral hospital in South India. Int J Pediatr Res.

[9] Kotwal YS, Jan FA, Yatoo GH, Kotwal S (2018). Neonatal profile and outcome of the neonates admitted in NICU: a hospital based prospective study. Int J Sci Res.

[10] Agrawal M, Bhatnagar K (2017). Maternal determinants affecting perinatal mortality: a multivariate statistical approach. Int J Reprod Contracept Obstet Gynecol.

[11] Jeganathan S, Ravikmar SA, Tamilmani A, Parameshwari P, Chinnarajalu AV, Kolkar YB (2017). Neonatal mortality of sick newborns admitted in a tertiary care teaching hospital in Tamil Nadu, South India. Int J Contemp Pediatr.

[12] Gogoi N (2018). Maternal and neonatal risk factors of low birth weight in Guwahati metro, Assam, northeast India. Acad J Ped Neonatol.

[13] Philip T, Pramod T (2018). A prospective study on neonatal outcome of preterm births and associated factors in a South Indian tertiary hospital setting. Int J Reprod Contracept Obstet Gynecol.

[14] Naskar N, Swain A, Das KN, Patnayak AB (2014). Maternal risk factors, complications and outcome of very low birth weight babies: prospective cohort study from a tertiary care centre in Odisha. J Neonatal Biol.

[15] Pengoria R, Agarwal M, Dayal R, Agarwal D, Singh M (2015). NICU admissions and neonatal outcome in high risk pregnancy. Ann Int Med Den Res.

